# A Survey to Determine Health Utility States After a Traumatic Brain Injury (TBI): Influence of a History of TBI on Disability Perceptions

**DOI:** 10.7759/cureus.70707

**Published:** 2024-10-02

**Authors:** Justin L Weppner, Isaiah Yim, Justin S Raucheisen, Melissa Martinez

**Affiliations:** 1 Physical Medicine and Rehabilitation, Virginia Tech Carilion School of Medicine, Roanoke, USA; 2 Internal Medicine, Virginia Tech Carilion School of Medicine, Roanoke, USA; 3 Physical Medicine and Rehabilitation, Carilion Clinic, Roanoke, USA

**Keywords:** brain trauma injury, decision-making in tbi, glasgow outcome scale-extended, traumatic brain injury, traumatic brain injury outcomes

## Abstract

Background

The Glasgow Outcome Scale-Extended (GOSE) has emerged as one of the most widely used outcome instruments for evaluating ongoing disability and recovery after traumatic brain injury (TBI). The influence of a personal history of TBI on disability perception and quality of life is not well understood. This study aimed to assess changes in health utility states using the GOSE among individuals with severe TBI and their caregivers compared to a general population group. We hypothesized that individuals with a history of TBI, either as patients or caregivers, would recognize health utility associated with a more severe disability than the general population group.

Methodology

This cross-sectional, observational study included 300 individuals with a history of severe TBI, 300 designated primary caregivers or family members, with 1:1 participation for each subject with severe TBI, and 300 participants from the general population. A computer-based survey was developed based on the GOSE. Participants assessed hypothetical scenarios representing one-year post-TBI outcomes using a standard gamble approach. The main measure for this study was participants’ perceptions of health-related quality of life and preferences for different GOSE health states following TBI.

Results

Of the 900 initial participants, 10 were excluded. Among the remaining 890 participants, lower GOSE states were rated to have lower health utilities. The general population group exhibited a notable decrease in health utility ratings from GOSE4 to GOSE3. Individuals with a history of severe TBI and their caregivers or family members experienced the most substantial decline in health utility ratings between GOSE3 and GOSE2. TBI and caregiver/family member status correlated with higher health utility ratings.

Conclusions

This study validated the use of the GOSE as a health utility metric and emphasized the subjective nature of acceptable outcomes. These findings underscore the need for considering personal experiences and preferences in decision-making regarding TBI care.

## Introduction

Traumatic brain injury (TBI) is a poorly understood global public health challenge. Each year, over 50 million people experience TBI worldwide, and it is estimated that approximately half of the global population have experienced at least one instance of TBI in their lifetime [1]. The Glasgow Outcome Scale-Extended (GOSE) has emerged as one of the most widely used outcome instruments for evaluating ongoing disability and recovery after TBI [2]. Ensuring interrater reliability and content validity is essential for providing consistent, fair, and evidence-based guidelines that protect vulnerable patients [3-5]. Patients with severe TBI may experience disorders of consciousness and be unable to make decisions regarding care [6]. Therefore, it is crucial to protect these patients from clinical nihilism, which can lead to self-fulfilling prophecies regarding mortality due to severe TBI [7]. Such prophecies can manifest through variations in withdrawal of care [8,9] and the use of prognostic indicators that have not been validated in previous research [10,11].

The disconnect between clinicians and caregivers or family members responsible for making decisions on behalf of patients with TBI is being increasingly recognized. A study conducted at a level 1 trauma center in the United States found that decision-making conflicts between clinicians and family members occurred in 57% of cases [12]. A recent study has revealed that a significant number of individuals with TBI and withdrawal of life-sustaining treatment might have survived and attained partial independence [13]. The unmet communication preferences of family members or physicians’ reluctance to provide numerical data due to concerns regarding promoting overconfidence can lead to mistrust and conflict. Therefore, studies are being conducted to develop a standardized protocol for clinical TBI management [14,15]. However, it is crucial to address potential differences in values between clinicians and family members or patients. Some clinicians’ tendency toward nihilism compared with prognostic models may explain the prevalence of disagreement with surrogates [7]. Establishing trust between clinicians and family members is vital for optimizing care and meeting the wishes of patients with TBI.

Health utility states are valuable tools for quantifying perceptions of TBI-related disability and evaluating individual preferences for measurable health-related outcomes such as the GOSE [16]. Health utility states summarize both positive and negative aspects of a health state as a single numerical value, facilitating comparisons in clinical research [17]. Determining GOSE health utilities after TBI is crucial for understanding the significant predictors of value judgments and improving the alignment between patient care and their values.

A model has been created to produce accurate and relevant utility estimates for GOS health states. This addresses shortcomings in current estimates, such as potential biases and limited generalizability. However, it is important to note that this model does not extend its application to GOSE scores [18]. While one study has examined public perceptions of disability outcomes following hypothetical TBI scenarios based on GOSE-based health utility, to our knowledge, no study has compared such perceptions between individuals with and without a history of TBI [17]. The impact of a personal history of TBI on the perception of disability and quality of life remains poorly understood. In this study, we aimed to measure changes in health utility states using the GOSE between individuals with severe TBI and their caregivers compared with the general population group and further validate the use of GOSE as a health utility metric for post-TBI outcomes. We hypothesized that individuals with a history of TBI, either as patients or caregivers, would recognize health utility associated with a more severe disability than the general population group.

## Materials and methods

This cross-sectional, observational study included 300 individuals with a history of severe TBI, 300 designated primary caregivers or family members, with 1:1 participation for each subject with severe TBI, and 300 participants from the general population. This study was reviewed and approved by the Institutional Review Board (IRB) of the institution where the research was conducted (approval number: 20-201). We affirm that this research has obtained ethical approval from the IRB and all participants provided written informed consent. The study was conducted in accordance with the Helsinki Declaration of 1975, as revised in 1983.

The study was conducted at an academic Level 1 Trauma Center between January 2021 and August 2022. Participants with TBIs and their family members/caregivers were enrolled in the study through an institutional-approved trauma registry. Participants with severe TBI were evaluated by a researcher who confirmed the diagnosis based on the study’s inclusion criteria. Caregivers of the participants were confirmed at the time of evaluation and enrollment in the study. The general population group was recruited through online convenience and chain referral sampling methods. This method facilitated the efficient collection of a diverse range of individuals from different backgrounds, age groups, and geographical locations.

The inclusion criteria for the severe TBI group required participants to be English-speaking and aged 18 years or older. They needed to have a documented severe TBI, which was defined by a Glasgow Coma Scale score of 3-8, a loss of consciousness lasting 24 hours or more, or post-traumatic amnesia lasting seven days or longer. Additionally, participants had to have the cognitive and physical abilities to complete the survey, the capacity to provide informed consent based on a structured interview and the MacArthur Competence Assessment Tool for Clinical Research, and a family member or caregiver available to participate in the study. The general population group was recruited as follows: English-speaking participants aged ≥18 years without a history of TBI or self-reported disability. The current GOSE scores of participants with TBI were assessed during screening for the study (Table 1). Measures such as Internet Protocol address tracking were implemented to prevent duplicate responses.

**Table 1:** The Glasgow Outcome Scale-Extended.

Score	Domain	Criteria
1	Death	
2	Vegetative State	
3	Lower Severe Disability	Completely dependent on others
4	Upper Severe Disability	Dependent on others for some activities
5	Lower Moderate Disability	Unable to return to work or participate in social activities
6	Upper Moderate Disability	Return to work at reduced capacity, reduced participation in social activities
7	Lower Good Recovery	Good recovery with minor social or mental deficits
8	Upper Good Recovery	No problems

Here, we employed an online computer-based survey based on structured interviews and scoring criteria of the original GOSE [16,19]. The survey offered detailed descriptions of the seven GOSE health states, highlighting the characteristics that determine an individual’s level of disability and functional status. Before conducting the survey, the included scenarios were independently reviewed and edited by three physicians with board certifications in brain injury medicine. The review process involved examination of the content, wording, and presentation of each scenario. The brain injury medicine physicians scrutinized the descriptions of the health states and factors influencing disability and functional status to ensure their alignment with the GOSE. They also assessed the clarity and comprehensibility of the scenarios. Three independent reviewers discussed the scenarios to enhance their reliability and consistency, shared their perspectives and insights, compared their evaluations, and worked collaboratively to achieve a consensus on the content and wording of each scenario.

Participants were given hypothetical computer-based scenarios depicting the outcomes one-year post-TBI and were shown each GOSE health state in sequence. After evaluating a health scenario, they moved on to the next, with no option to revisit previous scenarios. To mitigate carry-over effects, the order in which the GOSE health states were presented was randomized for each participant. Quality of life preferences for participants were evaluated through the utilization of the widely adopted standard gamble approach [17,20]. This method incorporates an element of risk to simulate inherent uncertainty in medical decision-making [21]. Here, participants were presented with a hypothetical choice between the following two options: (a) the certainty of continued life in a specific health state (GOSE2-8) or (b) taking a gamble with two possible outcomes, i.e., perfect health or immediate death. Initially, gambling probabilities were set at a balanced value of 50% for both immediate death and perfect health. Through a systematic adaptation process based on participant responses, the probabilities were adjusted until the point of indifference was reached, indicating the participants’ health preference for a specific GOSE state.

When participants identified a specific health state (e.g., GOSE2) as being worse than death, the decision-making process shifted to a choice between a certain death or a gamble involving perfect health and the health state perceived as worse than death. In cases where the health state was rated as worse than death, the point of indifference was represented by a negative value to encompass the notion of negative health utility.

Following completion of the quality of life evaluations for all seven scenarios, participants were requested to provide demographic information, including age, sex, race, education level, current employment status, and marital status; for participants with severe TBI, the time since injury and GOSE scores were recorded. To minimize the potential influence of stereotype threats on decision-making, demographic and personal questions were presented at the end of the survey rather than at the beginning [17,22]. The survey was administered separately to survivors of TBI and caregivers who were instructed not to discuss the survey until its completion.

Several criteria were applied to the survey responses to ensure data quality and reliability. Participants who either equally valued all health states or provided incomplete survey responses were excluded from the analysis. Equally valuing all health states was defined as assigning the same value to all seven GOSE scenarios. This exclusion criterion was implemented to ensure meaningful differentiation and assessment of the health states. If participants assigned identical values to all health states, it could suggest a lack of engagement with the survey or an inability to distinguish between different levels of health status, potentially introducing biases or inaccuracies into the analysis. This criterion was based on previous health utility studies that considered such responses to be implausible representations of individuals’ value judgments [17,23]. Incomplete responses were delineated as instances where participants granted informed consent and completed at least one GOSE scenario but failed to complete all seven scenarios. These incomplete responses were omitted from the analysis to uphold data consistency and integrity.

The following demographic characteristics of the final sample were analyzed: age, sex, race, education level, current employment status, marital status, the relationship of the family/caregiver with the participant in the family/caregiver group, and the time since the occurrence of TBI for participants with severe TBI. All statistical analyses were performed using SPSS version 26.0 (IBM Corp., Armonk, NY, USA).

## Results

**Figure 1:**
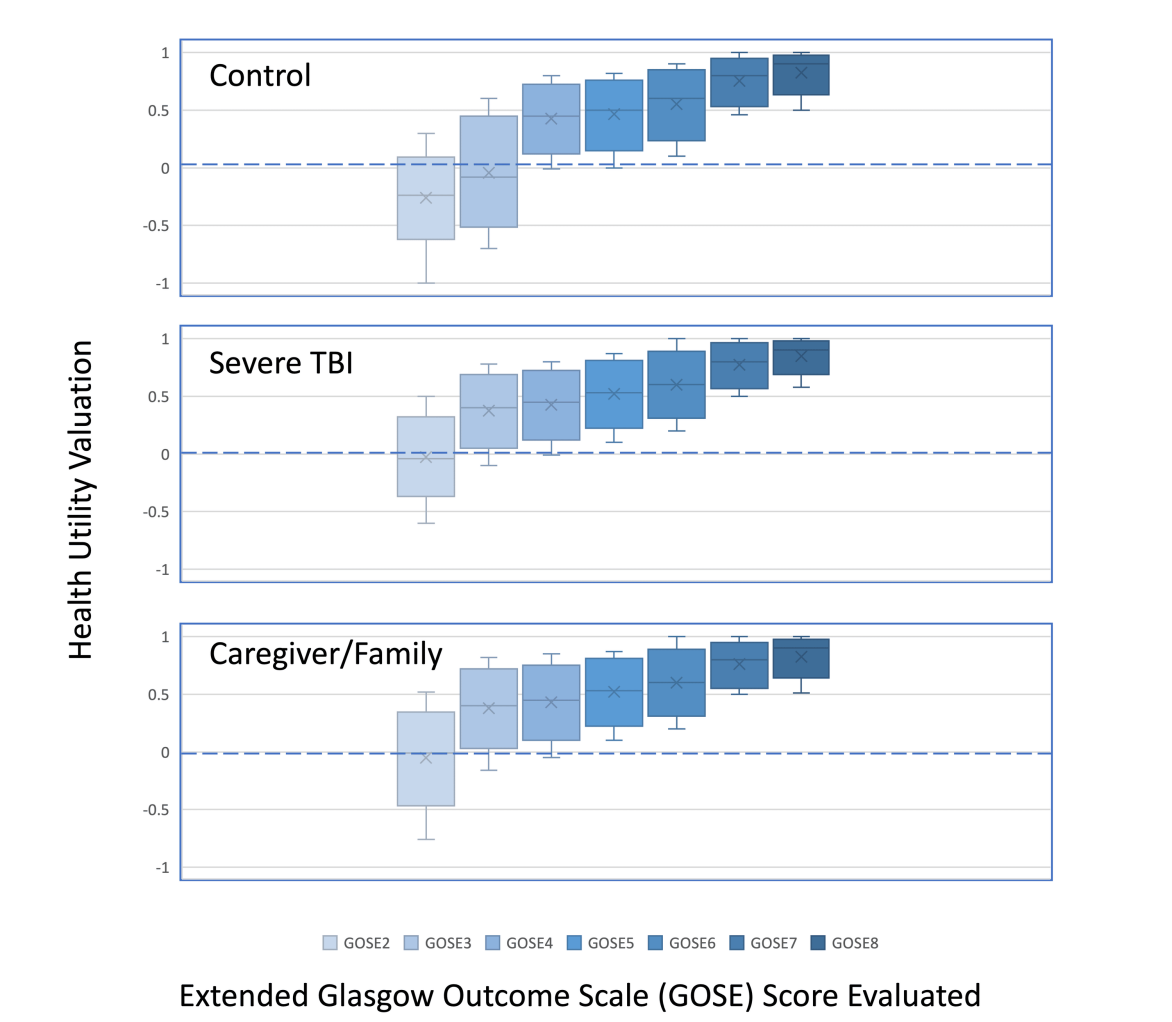
Unadjusted health utility valuations for different Glasgow Outcome Scale-Extended (GOSE) scores. This figure illustrates the unadjusted health utility evaluated by GOSE scores (ranging from 2 to 8) among the following three groups: 300 general population participants, 300 survivors of severe traumatic brain injury (TBI), and 300 caregivers/family members. Health utility ratings were obtained for scenarios one year after a hypothetical TBI. The box plots depict the distribution of health utility valuations for each GOSE score, presented in random order, and rated by 890 participants. The reference state, represented by the horizontal zero line, corresponds to death (GOSE1) and serves as a baseline for evaluating other health states. The participants did not evaluate the GOSE1 state by convention. A quality of life rating of 1 indicates perfect health, whereas a rating of 0 corresponds to death. Health states perceived to be worse than death are assigned a negative value. The box plots provide an overview of the variability and central tendency of health utility valuations for each GOSE score, highlighting the potential differences among the three participant groups. This figure allows for visual comparison and understanding of how different GOSE scores are associated with health utility valuations, providing insights into participants’ perspectives on their quality of life following a hypothetical TBI.

Of the 900 eligible participants, four (0.4%; two in the TBI group and two in the family/caregiver group) were excluded for completing only part of the seven GOSE surveys, and six (0.7%; one in the TBI group, two in the family/caregiver group, and three in the general population group) were excluded for providing equal valuations. Finally, responses from 890 participants who completed all seven GOSE scenarios were analyzed. The sample characteristics and demographic statistics are presented in Tables 2-4. Health utility medians showed that participants rated lower GOSE states as having lower utility. For the general population group, the greatest decline in health utility was observed from GOSE4 to GOSE3, while the median utilities for GOSE3 and GOSE2 were rated worse than death at -0.08 and -0.11, respectively. In contrast, participants with a history of severe TBI and their caregivers or family members showed no differences in the health utility of GOSE3-5. The greatest decline in health utility in the population with TBI was illustrated from GOSE3 to GOSE2. The median utility for GOSE2 was rated worse than death at -0.04 for those with severe TBI and -0.01 for caregivers and family members (Figure 1).

**Table 2:** Characteristics and descriptive statistics of participants. N represents the number of respondents. Data are presented at mean ± standard deviation (SD) or as a percentage (%).

Variables	N	Mean ± SD or %
Age (years)	890	38 ± 19
Sex		
Male	483	54.3%
Female	407	45.7%
Race		
White	759	85.3%
Black	49	5.5%
Asian	27	3%
Pacific Islander	2	0.2%
Other	24	2.7%
Not reported	29	3.3%
Education (years)		
12 or less	183	20.6%
13–14	139	15.6%
15–16	318	35.7%
17–18	129	14.5%
≥19	121	13.6%
Marital status		
Married	449	50.5%
Never married	294	33%
Separated	19	2.1%
Divorced	119	13.4%
Widowed	9	1%

**Table 3:** The amount of time since severe traumatic brain injury (TBI) and the corresponding Glasgow Outcome Scale-Extended (GOSE) scores assessed at the time of the survey. N represents the number of respondents. Data are presented at mean ± standard deviation (SD) or as a percentage (%).

Variable	N	Mean ± SD or %
Time since TBI (days)	297	422 ± 291
GOSE score		
3	8	2.7%
4	45	15.2%
5	40	13.5%
6	65	21.9%
7	79	26.6%
8	60	20.2%

**Table 4:** The percentage of caregivers/family members and their relationship with individuals with severe traumatic brain injury (TBI). N represents the number of respondents, with the percentage (%) of each caregiver/family member provided.

Relationship to participants with TBI	N	%
Spouse	146	49.3
Ex-spouse	16	5.4
Parent	87	29.4
Sibling	19	6.4
Adult child	17	5.8
Other family members	6	2
Friend	5	1.7

## Discussion

TBI is a significant global public health challenge that affects millions of people worldwide annually [1]. The GOSE is one of the most widely used outcome instruments for assessing ongoing disability and recovery after TBI. Health utility states are valuable tools for quantifying perceptions of TBI-related disabilities and evaluating individual preferences for measurable health-related outcomes. However, the impact of a history of TBI on perceptions of disability and quality of life remains poorly understood. Therefore, this study was conducted using the GOSE to measure changes in health utility between individuals with severe TBI and their caregivers compared with the general population and validate the use of GOSE as a health utility metric for post-TBI outcomes.

A total of 297 (99%) survivors of TBI and 296 (98.7%) family members or caregivers who completed the survey were randomly presented with a spectrum of disability outcomes one year after a hypothetical TBI. The findings revealed intriguing insights into the perception of health conditions and the impact of severe disability on the quality of life. More than 50% (N = 304) of the participants, comprising both TBI survivors and their family members or caregivers, believed that being in a vegetative state constitutes a health condition worse than death. However, it is worth noting that the majority of both survivors of TBI and their family members or caregivers did not consider requiring 24/7 supervision for severe disability after TBI to be worse than death. This suggests that while the challenges and restrictions imposed by severe disability are recognized, they do not necessarily equate to a perception that life is devoid of value or purpose.

In contrast, when examining the responses of the 297 (99.5%) participants from the general population who completed the survey, it was observed that over 75% (N = 224) regarded being in a vegetative state as a health condition that was worse than death. This stark contrast in perception between general population individuals and those directly affected by TBI highlights the significant impact of first-hand experiences on one’s understanding of the condition. Similarly, more than 50% (N = 162) of the general population group considered requiring 24/7 supervision for severe disability after TBI to be worse than death. This suggests that the general population tends to have a bleaker outlook on the implications of severe disability than individuals who have experienced TBI or are closely involved in caring for someone with TBI.

Our analysis indicated that individuals with a history of severe TBI and their caregivers or family members did not demonstrate significant differences in health utility in GOSE3-5. The most substantial decline in health utility among the population with TBI and their caregivers or family members was observed when transitioning from a lower severe disability (GOSE3) to a vegetative state (GOSE2). In contrast, the general population group exhibited the greatest change in the mean health utility score when moving from the upper severe disability (GOSE4) to the lower severe disability (GOSE3) states, which was in line with a previous study exploring public perceptions of disability [17].

Typically, TBI researchers consider GOSE health valuations, but there is a floor effect observed among the lowest GOSE outcomes, with GOSE3 being the cut-off for a poor outcome in previous studies [24,25]. This cut-off was consistent with the values observed in our general population group. However, individuals who experienced severe TBI and their caregivers assigned higher health utility values to states of lower functionality. Many did not view the GOSE3 state as a condition that was worse than death.

This study highlights that acceptable GOSE score outcomes following TBI are highly subjective and influenced by personal experience. This suggests that individuals who have not experienced severe TBI should avoid adopting a nihilistic view and should refrain from imposing bias on patients and their families. However, it is crucial to recognize that determining an acceptable outcome is a personal choice. Healthcare professionals and decision-makers should strive to understand and respect the preferences and values of patients and their families without projecting their own beliefs regarding the situation. By doing so, they can ensure that care and decisions align with an individual’s unique circumstances and needs.

Despite the valuable insights provided by this study, several limitations should be acknowledged. The study relied on self-reported measures and hypothetical scenarios, which may have introduced recall bias and subjective interpretations. The participants’ responses to hypothetical scenarios may not fully reflect their actual preferences and experiences in real-life situations. Another limitation of this study was that participants with a history of severe TBI were required to have cognitive abilities to provide informed consent. Additionally, the participants were required to have a caregiver or family member who participated in the study. This inclusion criterion may have excluded individuals with more severe impairments or disabilities, potentially limiting the generalizability of our findings to the entire population of individuals with severe TBI. Additionally, the study predominantly included English-speaking participants, which may limit the generalizability of our findings to other populations with diverse linguistic and cultural backgrounds. Moreover, this study focused on participants with severe TBI, which may not have fully captured the perspectives and experiences of individuals with mild or moderate TBI. The exclusion of these milder cases limits a comprehensive understanding of the impact of TBI across the entire spectrum of severity. Another limitation of this study is the potential bias introduced by online convenience and chain referral sampling methods, which may not provide a fully representative sample of the broader population. Furthermore, this study relied on a single outcome measure, the GOSE, to assess health utility. Although the GOSE is widely used and validated, it may not capture the full range of dimensions and complexities associated with TBI-related disability and quality of life. The wide scope of GOSE categories, exemplified by GOSE3 that involves individuals in minimally conscious states or those who can be left alone for periods less than eight hours, underscores the sensitivity of ratings to variations in scenario wording. Nevertheless, it is important to acknowledge that this inherent limitation arises when using GOSE for outcome assessments. Future studies should incorporate additional outcome measures for a more comprehensive evaluation of health utility and disability outcomes.

To advance the field of TBI effectiveness research, it is important to expand the development of health utilities beyond current measures. This study highlights the significance of considering individual preferences in the shared medical decision-making process regarding potential disability outcomes following TBI. Furthermore, there is a need to explore how physicians perceive and assign health utility values to different health states using the GOSE. Gaining insights into the perspectives of healthcare providers can contribute to the development of comprehensive guidelines and prognostic aids that are better aligned with patient preferences. By considering physicians’ perspectives, it may be possible to bridge the gap between healthcare professionals and patients/family members, promoting a more holistic and patient-centered approach toward TBI management. The findings of this study supported our hypothesis that individuals with a history of TBI, including patients and their family members, perceive health utility in lower GOSE scores compared to the general population. This highlights the need to consider the impact of TBI on health-related quality of life.

## Conclusions

This study examined the health utility and perceptions of disability in individuals with TBI and their caregivers or family members compared to a general population group. These findings highlight the subjective nature of acceptable outcomes and the influence of personal experiences on health valuations. This study emphasized the importance of understanding and respecting individual preferences and values when making decisions regarding TBI care. While the study provides valuable insights, it also has several limitations, such as reliance on self-reported measures and a sample comprising predominantly English-speaking individuals. Nonetheless, the findings of this study have the potential to guide future research and enhance the understanding and management of TBI, leading to improved patient outcomes and quality of life.
